# A Desirable Result

**Published:** 1879-04

**Authors:** 


### A DESIRABLE RESULT.

In an editorial note appended to the description of the case given in the March number of the REGISTER, page one hundred and twenty-eight, it is stated that at that time, March 9, the catamenial discharge had not been established in the normal way.

A letter is just at hand, March 27, saying that menstruation has taken place fully and in the natural order, thus removing any apprehension of the return of the disease in the mouth, which appeared before the operation to be of so serious a character.

